# Antioxidant Activity of Leaves and Fruits of Cultivated Conifers in Iran

**DOI:** 10.17795/jjnpp-9670

**Published:** 2013-07-20

**Authors:** Sayed Ahmad Emami, Ali Shahani, Mohammad Hassanzadeh Khayyat

**Affiliations:** 1 Department of Pharmacognosy, School of Pharmacy, Mashhad University of Medical Sciences, Mashhad, IR Iran; 2 Pharmaceutical Sciences Research Center, Department of Medicinal Chemistry, School of Pharmacy, Mashhad University of Medical Sciences, Mashhad, IR Iran

**Keywords:** Cupressaceae, Pinus, Antioxidants

## Abstract

**Background:**

Use of antioxidants is a means of reducing rancidity of fats and oils in food stuffs. The commercial synthetic antioxidants in food industry have been suspected to cause negative health effects. Therefore as alternatives, there is a strong need in finding new effective and safe antioxidants from natural sources to prevent deterioration of foods, drugs and cosmetics.

**Objectives:**

In order to investigate the antioxidant activity from natural sources as alternatives of commercial antioxidants, the antioxidant activity of the extracts of leaves and fruits of six different species of Iranian common conifers: *Cupressus arizonica*, *Pinus halepensis*, *Pinus nigra*, *Pinus brutia* var. *elderica*, *Pinus wallichiana* and *Cedrus deodara* were investigated in the current study.

**Materials and Methods:**

The leaves and fruits of these plants were collected from different areas of the country. Methanol extracts of leaves and fruits of Iranian common conifers (six species) were prepared. Antioxidant activity of the samples was measured using ferric thiocyante (FTC) and thiobarbituric acid (TBA) tests.

**Results:**

The results of the study, using the two methods, showed that all methanol extracts of leaves and fruits of the six species possessed antioxidant activity. The antioxidant activity of the samples was compared with the antioxidant activities of butylatedhydroxytoluene (BHT), as a synthetic antioxidant and α-tocopherol as a natural antioxidant. Methanol extractions of *Pinus *spp. leaves and fruits showed the highest antioxidant activity (quite higher than α- tocopherol). Among the extracts examined, the leaves of *P. halpensis* methanol extract showed the lowest activity.

**Conclusions:**

At this stage it can be concluded that methanol extracts of these plants can be considered as strong antioxidant agents. However more investigation is necessary in order to evaluate the antioxidant activity of the components separate from each extracted sample using several different antioxidant methods.

## 1. Background

It is generally accepted that free radicals play an important role in the development of tissue damage and pathological events (1-3). Molecular oxygen readily oxidizes lipids containing polyunsaturated fatty acids. This kind of oxidation is caused by a free radical chain mechanism (2, 4). Lipid peroxidation can lead to aging, and many other diseases like coronary heart disease, diabetes mellitus, rheumatic disease and liver disorders. They may also cause multiple sclerosis, Parkinson’s disease and many others (5-13). Antioxidants are capable of stabilizing or deactivating free radicals before they attack cells. Therefore the need for antioxidants becomes more critical with increased exposure to free radicals. Pollution, cigarette, smoke, drugs, illnesses and even exercise can increase free radical exposure (13). There are many investigations related to finding antioxidative compounds to be used as radical scavengers in living organisms and also to prolong the shelf life of food products. As with other synthetic food additives, the popular synthetic antioxidants (butylatedhydroxy toluene BHT, butylatedhydroxy anisole BHA, propyl galate PG and tertiary butyl hydroquinone TBHQ) in food industry, to delay or prevent oxidation of the substrate have been criticized mainly due to possible toxic effects or promoting negative health effects (14). Therefore, there is an increasing world-wide interest in finding new effective and safe antioxidants from natural sources as alternatives of commercial antioxidants (14-20). Antioxidants from natural sources can serve as leading compounds for the development of new drugs with the prospect of improving the treatment of various disorders. They can also be used in food and pharmaceutical technology as an alternative to the synthetic compounds. Native conifers are a small group of the flora of Iran (seven species from 8000 species). The following conifers are also cultivated in Iran (Iranian common conifers): *Cupressus arizonica *Greene, *Pinus halepensis *Mill., *Pinus nigra* J. F. Arnold, *Pinus brutia* var. *elderica* (Medw.) Salisb. [Syn: *Pinus elderica *Medw.], *Pinus wallichiana* A. B. Jacks. and *Cedrus deodara* (Roxb.) Lous. [Syn: *Pinus deodara *Roxb.]. They are evergreen and aromatic trees. These plants are widely spread and grow in different parts of many countries including Iran. Their Persian names are SarveNoghreei and SarveSimin, KajHalab, KajSyah, Kaj Tehran, KajGeryan and Divdar respectively (21-24). Most of these trees are medicinal plants and their dried leaves and fruits are used to treat various diseases like asthma, stomachic, tuberculosis, liver disease, rheumatism, dermatitis and inflammation. They were also used for their bronchodilation activities, antibacterial activity, alleviating nausea, anthelmintic, astringent, digestive, expectorant, fungicidal and antioxidant activity (25-28).

## 2. Objectives

Several researches have been done regarding antioxidant activity of various species of conifers in order to investigate the antioxidant activity from natural sources as alternatives of commercial antioxidants (28-39). However there are no published reports on antioxidant properties of the crude extract (methanol) obtained from leaves and fruits of Iranian common conifers. Therefore in the present investigation antioxidant activity of the crude extract (methanol) of common Iranian conifers (six species); *Cupressusarizonica*, *Pinus halepensis*, *Pinus nigra*, *Pinus brutia* var. *elderica*, *Pinus wallichiana* and *Cedrus deodara* was evaluated.

## 3. Materials and Methods

### 3.1. Materials

All chemicals were purchased from Sigma (Sigma Aldrich GmbH. Sternheim), Merck (Darmstadt) and Roth (Karlsruhe) in Germany.

### 3.2. Plant Materials

*Cupressus arizonica*, *Pinus halepensis*, *Pinus nigra* and *Pinusbrutia* var. *elderica* fruits and leaves, all were collected from Ferdowsi University Pardis area at height of 933 m (located in Mashhad, RazaviKhorasan province, north east of Iran), *Pinus wallichiana* was collected from Park Melat at height of 933 m (located in Mashhad, Khorasan Razavi province, north east of Iran) and *Cedrus deodara* was collected from Nowshar Ecological Garden, at height of 23m (located in Nowshar, Mazandaran province, north of Iran). All the plants were collected on the beginning of September 2011 and were identified in Nowshahr Ecological Garden by Mr. H. Zare. To avoid any destruction in the chemical components of the collected materials they were stored at -20˚C (40).

### 3.3. Extraction of the Samples

Individual fruits and fresh leaves of each plant (200 g fresh wt.) were chopped in small pieces separately and then crushed into powder by a blender. The pure methanol (300 mL) was used to macerate each powdered sample for 24 hours. The macerated powder sample was percolated using about one litter of methanol. Then the methanol extracts were concentrated under reduced pressure at 50˚C to be dried. All the extracted samples were stored at -20˚C until their antioxidant activity evaluated.

### 3.4. Antioxidant Assays

Several reports have been published on evaluation of the antioxidant activity of the extract of various species of conifers (28-39). In the current study the antioxidant activity of the methanol extract (final concentration 0.02% w/v) of leaves and fruits of Iranian common conifers (six species) were investigated using two different antioxidant assays; Ferric thiocyanate (FTC) and thiobarbituric acid (TBA) methods (41).

#### 3.4.1. Ferric Thiocyanate Method

In FTC method 4 mg of Different extracts and/or standards (4 mg; BHT and vitamin E) was placed in a vial with a screw cap and then mixed with four mL of absolute ethanol. From 2.52% linoleic acid solution in absolute ethanol, 4.1 mL was added to the mixture as well as eight mL of 0.05 M phosphate buffer (pH = 7) and 3.9 mL of distilled water. The vial was placed in an incubator at 40˚C in the dark. To 0.1 mL of this solution 9.7 mL of 75% ethanol (v/v) and 0.1 mL of 30% ammonium thiocyanate were added. After three minutes, 0.1 mL of 0.02 M ferrous chloride in 3.5% hydrochloric acid was added to the reaction mixture. The red color absorbance was determined every 24 hours at 500 nm until the day after the control sample reached its maximum absorbance’s. As positive controls Vitamin E and BHT were used in these experiments. A mixture having no plant sample was used as a negative control.Both positive controls and negative control were subjected to the same procedures (41-43).

#### 3.4.2. Thiobarbituric Acid Method

Another method used to measure the extent of antioxidant activity was thiobarbituric acid (TBA) method. In this method two mL of 20% trichloroacetic acid and two mL of 0.67% 2-thiobarbituric acid solutions were added to two mL of the mixtures containing the sample prepared in the FTC method. The mixture was placed in a boiling water bath and centrifuged at 3000 rpm for 20 min after cooling down to room temperature. On the final day of the assay the absorbance of the supernatant (at 532 nm) was the base of the antioxidant activity calculation. In both FTC and TBA Methods the following formulae was used to estimate the percentage of antioxidant activity of the samples:

AI, % = 100 × (A_0_-A)/A_0_

In this formulae, A_0_ indicates the absorbance of the control sample where the reaction contains no test compound. The absorbance of the control samples were taken as 100% lipid peroxidation. To achieve reliable antioxidants activity results, for each sample, average results of repeating for five times was used for calculation. SPSS software was employed to conduct a one way ANOVA to compare the antioxidant activity of the extracts and positive controls at P ≤ 0.05 level of significance .

## 4. Results

**Figure 1.**
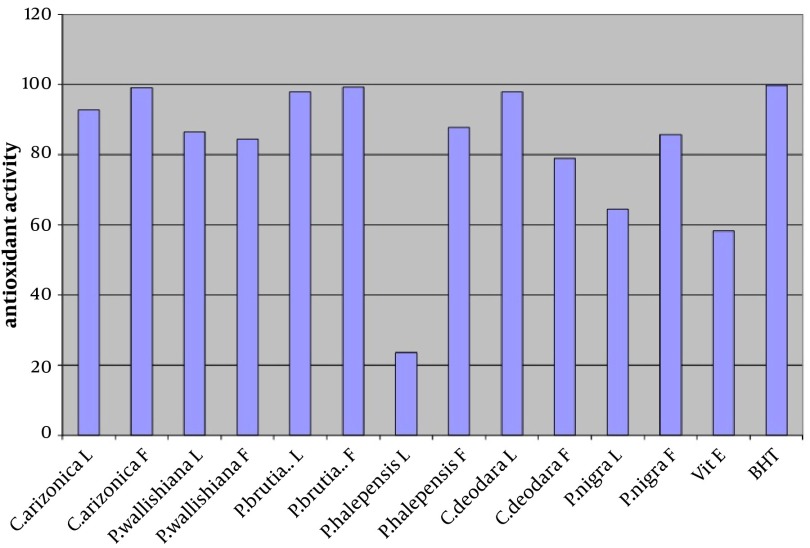
Antioxidant Activity of Methanol Extracts of Leaves (L) and Fruits (F) of Six Different Species of Common Iranian Conifers (Final Concentration 0.02% w/v) Measured Using FTC Method

**Figure 2.**
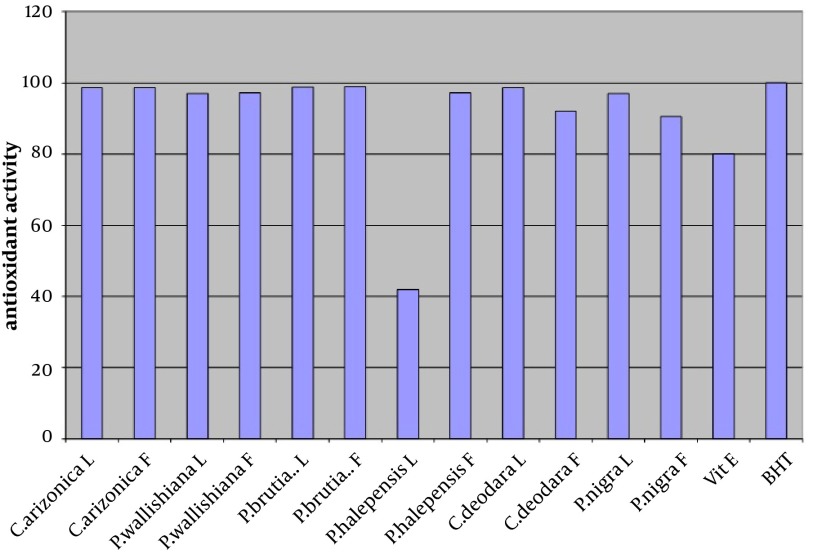
Antioxidant Activity of Methanol Extracts of Leaves (L) and Fruits (F) of Six Different Species of Common Iranian Conifers (Final Concentration 0.02% w/v) Measured Using TBA Method

The antioxidant activity of the crude methanol extract (at concentration of 0.02%) of leaves and fruits of six different species of common Iranian conifers (twelve samples); *Cupressus arizonica*, *Pinus halepensis*, *Pinus nigra*, *Pinus brutia* var. *elderica*, *Pinus wallichiana* and *Cedrus deodara* was evaluated in the current study by using FTC and TBA methods (Figures 1 and 2 respectively). 

In both methods while using one sample without antioxidant activity as control sample, vitamin E and Butyl Hexatoluene (BTH) (at concentration of 0.02%) were used as standards. Nearly all the methanol extracts of fruits and leaves of common Iranian conifers (six different species) showed strong antioxidant activity when both FTC and TBA methods were used. The methanol extract of the fruits of *P. brutia* var. *elderica* showed the highest antioxidant activity while the *P. halpensis* methanol extraction possessed the lowest antioxidant activity when the FTC and TBA methods were used.

## 5. Discussion

Free radicals play an important role in the development of tissue damage and pathological events (1-3). There is enough evidence indicating that indigenous antioxidants may be useful in preventing the deleterious consequences of oxidative stress. Therefore the need for antioxidants becomes more critical with increased exposure to free radicals (13). Since using synthetic antioxidant compounds to delay or prevent oxidation of the substrate, has been criticized, mainly due to possible toxic effects or promoting negative health effects (14), therefore there is an increasing interest in finding new effective and safe antioxidants from natural sources like herbs and medicinal plants as alternatives of commercial antioxidants in order to avoid destructive changes in cosmetics, drugs, and foods (16-20, 44). However there are many published reports evaluating the antioxidant activity of the extracts and the essential oils of various plants. A potent broad spectrum scavenger of these extracts and oils may serve as a possible preventive intervention for free radical mediated cellular damage and diseases (44). There are several reports on the evaluation of antioxidant activities of extracts and essential oils of *Pinus* species mainly *P. halepensis* using different antioxidant activity tests. Results indicated that the extracts and essential oils of these plants usually showed a strong activity in these antioxidant assays (28-39). But there is no report on evaluation of antioxidant effect of the methanol extracts obtained from leaves and fruits of Iranian common conifers. The antioxidant activity of the crude methanol extract of the leaves and fruits of six different species of common Iranian conifers was evaluated in the current study by FTC and TBA methods (Figures 1 and 2 respectively). In order to estimate the amount of peroxide formed during the initial stage of linoleic acid peroxidation the, FTC method was used. In this method to form a reddish ferric chloride pigment, the peroxide reacts with ferrous chloride. During the reaction as the antioxidant activity increases the concentration of peroxide decreases. The absorbance values of control sample showed increase from the day 1 and reached the top on day five and dropped on day 6. Increase in the level of melonaldehyde compounds from linoleic acid oxidation, which is not stable, is the reason of the reduction in the amount of absorbance. However the absorbance of the final day was based for calculation of sample antioxidant activity. Subsequently at a later stage of lipid oxidation, peroxide decomposes to form carbonyl compounds that are measured by the TBA method. In TBA method the absorbance of the final day was based to calculate the antioxidant activity of the samples which showed total peroxide values produced by oxidation of linoleic acid. Lower level of antioxidant in the samples shows higher values in absorbance. Vitamin E and Butyl Hexatoluene (BTH) (at concentration of 0.02%) were used as standard in both methods and one sample without antioxidant activity was used as control. The values obtained from the control samples were taken for 100% lipid peroxidation. Based on the results, both in the FTC and TBA methods, nearly all the methanol extracts of leaves and fruits of six different species of common Iranian conifers possessed strong antioxidant activity (low absorbance values), when compared to the positive controls vitamin E (a natural antioxidant) and BHT (a synthetic antioxidant). The pattern of activity was very similar for both methods. Compared with the BHT activity, different extracts obtained from different parts of plants exhibited very strong antioxidants activity within the range of 90 - 99% (the extract of fruits of *Pinus brutia *var. *elderica *showed the highest activity, 98.97%) by the FTC method (except for the extract of leaves of *Pinus halepens *which was 42.02%). Figure 1 Statistical analysis showed no significant differences between antioxidant activity of the methanol extracts of leaves and fruits of *Pinus brutia* var. *elderica*, *Cupressus arizonica* and *Pinus wallichiana* and methanol extract of leaves of *Cedrus deodara* fruits of *Pinus halepensis* and fruits of *Pinus nigra* (P < 0.05). In the TBA method, all the extracts also showed strong antioxidant activity when compared with those of the BHT activity. The extracts activity ranged between 65 - 99% where the extract of fruits of *Pinus brutia* var. *elderica* showed the highest activity (99.30%) and the extract of leaves of *Pinus halepens* showed the lowest activity (23.56%), Figure 2 Results obtained from TBA method showed no significant differences between antioxidant activity of the methanol extracts of leaves and fruits of *Pinus brutia* var. *elderica*, fruits of *Cupressus arizonica* and leaves of *Cedrus deodara* (P < 0.05). However among the examined extracts the leaves of *P. halpensis* methanol extraction possessed the lowest antioxidant activity in both FTC and TBA methods. Although antioxidant activities of extracts of *Pinus* species (mainly *P. halepensis*) using different antioxidant activity tests have been investigated but there is no report on evaluation of antioxidant effect of the methanol extracts obtained from leaves and fruits of Iranian common conifers. In the current study the methanol extract of leaves and fruits of six different species of common Iranian conifers; *C. arizonica*, *P. halepensis*, *P. nigra*, *P. brutia* var. *elderica*, *P. wallichiana* and *C. deodara* showed strong antioxidant activity when they were tested by FTC and TBA methods. To conclude the results of the present investigation, it is necessary to determine the antioxidant activity of the component of each extracted sample separately using different antioxidant methods. However the results of the present study showed that the methanol extracts of investigated common Iranian conifers can be considered as strong antioxidant agents.
